# A Speech-Based Mobile Screening Tool for Mild Cognitive Impairment: Technical Performance and User Engagement Evaluation

**DOI:** 10.3390/bioengineering12020108

**Published:** 2025-01-24

**Authors:** Rukiye Ruzi, Yue Pan, Menwa Lawrence Ng, Rongfeng Su, Lan Wang, Jianwu Dang, Liwei Liu, Nan Yan

**Affiliations:** 1 Guangdong-Hong Kong-Macao Joint Laboratory of Human–Machine Intelligence-Synergy Systems, Shenzhen Institutes of Advanced Technology, Chinese Academy of Sciences, Shenzhen 518055, China; rkym.rouzi@siat.ac.cn (R.R.); rf.su@siat.ac.cn (R.S.); lan.wang@siat.ac.cn (L.W.); jdang@jaist.ac.jp (J.D.); 2 Advanced Computing and Storage Laboratory, Central Research Institute, 2012 Laboratories, Huawei Technologies Co., Ltd., Nanjing 210012, China; panyue32@huawei.com; 3 Speech Science Laboratory, Faculty of Education, University of Hong Kong, Hong Kong SAR, China; manwa@hku.hk

**Keywords:** mobile health applications, mild cognitive impairment, MCI detection, automatic screening, user engagement

## Abstract

Traditional screening methods for Mild Cognitive Impairment (MCI) face limitations in accessibility and scalability. To address this, we developed and validated a speech-based automatic screening app implementing three speech–language tasks with user-centered design and server–client architecture. The app integrates automated speech processing and SVM classifiers for MCI detection. Functionality validation included comparison with manual assessment and testing in real-world settings (*n* = 12), with user engagement evaluated separately (*n* = 22). The app showed comparable performance with manual assessment (F1 = 0.93 vs. 0.95) and maintained reliability in real-world settings (F1 = 0.86). Task engagement significantly influenced speech patterns: users rating tasks as “most interesting” produced more speech content (*p* < 0.05), though behavioral observations showed consistent cognitive processing across perception groups. User engagement analysis revealed high technology acceptance (86%) across educational backgrounds, with daily cognitive exercise habits significantly predicting task benefit perception (H = 9.385, *p* < 0.01). Notably, perceived task difficulty showed no significant correlation with cognitive performance (*p* = 0.119), suggesting the system’s accessibility to users of varying abilities. While preliminary, the mobile app demonstrated both robust assessment capabilities and sustained user engagement, suggesting the potential viability of widespread cognitive screening in the geriatric population.

## 1. Introduction

### 1.1. Background

Mild Cognitive Impairment (MCI) is widely recognized as a transitional stage between the cognitive changes associated with normal aging and the more severe cognitive decline commonly found in Alzheimer’s disease (AD) [1]. Early detection of MCI is crucially important as it provides an opportunity for accurate diagnosis and timely intervention that can effectively slow down or prevent disease progression, reducing the risk of Alzheimer’s disease [2,3,4]. MCI is believed to arise from a combination of various factors, including age, genetics, lifestyle, and overall health conditions [5]. Individuals with MCI experience a mild decline in cognitive abilities, including memory, language, and cognitive processing such as reasoning and problem solving. However, unlike AD, MCI symptoms are often subtle and difficult for individuals and even their family members to notice. Despite the high prevalence of MCI in China, of 15–50% within the senior population (aged 60+) [6], diagnosis and treatment rates remain alarmingly low [7], emphasizing the urgent need for scalable screening solutions.

Traditional diagnostic approaches of MCI present some limitations in their accessibility and scalability. Image-based methods, such as magnetic resonance imaging (MRI) [8] and positron emission tomography (PET) [9,10], offer high diagnostic accuracy, but they are expensive and often not easily accessible. Yet, cerebrospinal fluid (CSF)-based methods [11,12,13] are invasive procedures that carry potential risks to the patients. Cognitive behavioral tests such as the Montreal Cognitive Assessment (MoCA) [14] and the Mini-Mental State Examination (MMSE) [15], while widely used, have been shown to have limited sensitivity and consistency for early detection [16,17,18].

Currently, several speech and language-based screening solutions have been developed for MCI assessment, as speech and language analysis can effectively characterize language disorders in neurodegenerative diseases [19,20], and it shows promise in detecting changes in language and speech patterns and exhibits the ability to identify early-stage cognitive decline [21,22,23,24]. Such solutions for MCI screening mainly comprise systems utilizing PC screens or web platforms as interfaces, such as the “CognoSpeak” system [25], intelligent virtual agents [26,27], the MARC rapid screening tool [28], and the Philips IntelliSpace Cognition digital test battery [29]. While these systems offer advantages over traditional assessments, most of these existing solutions are designed for use on PC screens, web platforms, or virtual agents, which may limit their accessibility and remote usage potential for widespread adoption.

With increased accessibility, mobile screening tools for cognitive impairment hold significant promise in healthcare by enabling early detection and intervention, even in remote or underserved areas—crucial for effective treatment and management of conditions like dementia [30,31]. Compared to traditional screening methods, mobile solutions are highly cost-effective [31,32] and more efficient, with a reduced need for manual administration and potential human biases [32,33]. Additionally, mobile apps can leverage speech language technologies to enable natural human–machine interaction, fostering greater acceptance, particularly among the elderly population who may face barriers in adopting new technologies [30,34].

While mobile applications for MCI screening demonstrate encouraging diagnostic accuracy, with Under the Curve (AUC) values up to 0.838 [18,35,36], and enable home-based cognitive function monitoring [37], their successful implementation depends heavily on user engagement. This aspect is particularly crucial as these applications primarily target older adults, who may face aging-related barriers to digital health technology adoption [38].

Strong user engagement is essential for achieving optimal effectiveness and the clinical impact of mobile mental health applications [39]. Understanding user engagement patterns and barriers is invaluable for developing user-friendly designs that promote sustained use and adherence. Therefore, our study extends beyond technical validation to include comprehensive user engagement analysis, aiming to optimize the app’s potential for early detection and intervention in the elderly population.

### 1.2. Objectives

The present study builds upon our previous work on discriminating MCI status using multiple spoken tasks [40] and addresses the gaps in accessibility, scalability, and user engagement by developing an automatic MCI screening app designed specifically for the geriatric population. It was assumed that an optimized and reliable app that is also user friendly and engaging is more likely to be accepted and used consistently, leading to more comprehensive cognitive assessments and helping to promote early detection of MCI on a large scale. With that, two interconnected objectives were addressed. First, the technical performance and functionality of the app’s performance in both controlled and real-world settings were validated, in order to establish its reliability and validity as an accessible and scalable tool for early detection of MCI. Second, we evaluated the ability of the proposed app to maintain user engagement, a critical factor in the success and impact of mobile health applications [39]. A comprehensive user engagement analysis that examined both subjective user evaluations and objective behavioral observations was conducted. This was to demonstrate the effectiveness in fostering user interaction and acceptance in MCI screening of the app. The specific research questions and hypotheses guiding this user engagement study are detailed in the next section.

### 1.3. Research Questions and Hypotheses for User Engagement Study

While the speech–language-based mobile app demonstrates strong potential for automated MCI screening, its effectiveness and clinical utility heavily rely on users’ sustained engagement and interaction. Prior research has emphasized the critical role of user engagement in determining the success and impact of mobile health applications, particularly among older adults who may face unique challenges in adopting new technologies [39]. To investigate this, a comprehensive user engagement analysis was conducted to explore the following three key research questions:

RQ1: Is there a correlation between users’ perceived difficulty of the screening process and their actual cognitive performance?

**Hypothesis 1.**  *It is hypothesized that users who perceive the app as more difficult to use will demonstrate lower cognitive performance on the MCI screening tasks*.

RQ2: How do users’ task-specific evaluations relate to their engagement and subsequent speech production within the app?

**Hypothesis 2.**  *It is expected that users who find a task more interesting or engaging will interact longer, and produce more speech within that task*.

RQ3: Do users’ daily habits regarding cognitive exercises influence their perceptions of the app’s potential usefulness and their willingness to embrace such technologies?

**Hypothesis 3.**  *It is anticipated that users who regularly engage in cognitive stimulation activities will show greater receptivity to the app’s benefits and demonstrate higher technology acceptance*.

## 2. Methods

### 2.1. Mobile App Design

#### 2.1.1. App Architecture and Development Framework

**Figure 1:**
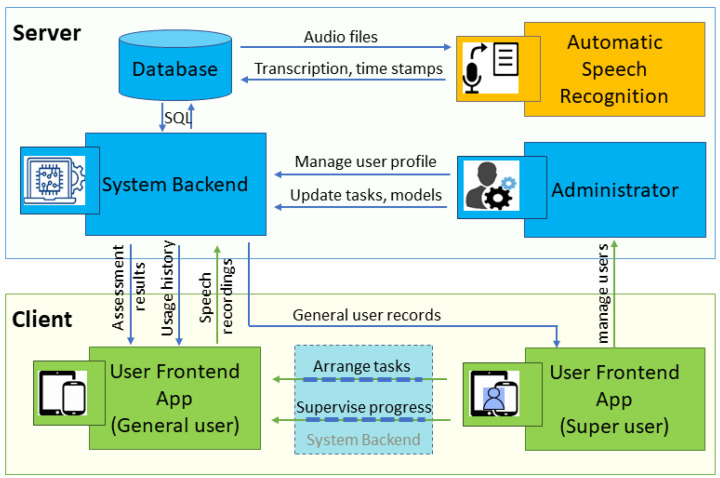
System architecture of our app. Color Styles (boxes and arrows): Blue:Server-side components, server-to-user data/control flow; Green: Client-side applications, user-to-server data/control flow; Yellow box: Processing component; Dotted boxes/lines: Features under development.

The MCI assessment application employs a client–server architecture to optimize resource allocation and ensure scalability (see Figure 1). The design separates the resource-demanding tasks and sensitive data handling from the user interface, improving both performance and security while enabling easier future updates and scalability.

The server hosts computational resources and data storage for the app. It includes a system backend that processes algorithms related to assessment models, a database for processed data and assessment results, and an Automatic Speech Recognition (ASR) module to process and recognize audio inputs and generate corresponding transcriptions as well as time stamps. An administrative function is also included for centralized user profile management, task updates, and model refinements.

**Figure 2:**
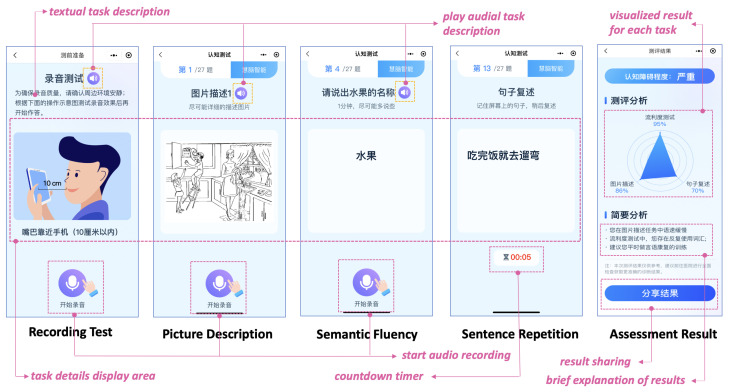
Screenshots of main user interface (UI) of the app. The screen will automatically switch to landscape orientation during the Picture Description task for better viewing. Detailed descriptions of the tasks are provided in Supplementary Material SA.

The client app primarily serves as the user interface, designed to facilitate accessibility for the geriatric population. Two distinct frontend interfaces have been designed as follows: A general user interface (see Figure 2) developed for potential users with MCI, which displays speech tasks, collects user inputs, and communicates with the server for data processing and result retrieval. A super user interface (under development) for specialists, community workers, or caregivers to manage assessments of users under their care. Both interfaces allow access to usage history and assessment records.

#### 2.1.2. Security Considerations and Data Protection

The application implements comprehensive security measures to protect sensitive healthcare data and prevent potential security risks. To address voice spoofing concerns highlighted in recent research [41], speech recordings are not downloadable by users without administrator permission. Personal information is encrypted in accordance with healthcare data protection standards, and unnecessary permissions (location, biometric authentication, email) are omitted to minimize security vulnerabilities [42]. The application follows strict security guidelines, requiring explicit user consent for microphone activation and avoiding non-essential access requirements [43].

#### 2.1.3. User-Centered Design Principles

The development of the MCI assessment application was guided by user-centered design principles, specifically optimized for elderly users to ensure ease of use. It incorporated three key aspects: interface accessibility, interaction convenience, and user-centric output.

Interface Accessibility: The interface design (see Figure 2) was carefully tailored to incorporate visual elements prioritized for older adults. Firstly, typography was given significant attention by implementing words of larger fonts with optimal contrast ratios to accommodate the potential visual impairments common in elderly users. Secondly, spatial organization was enhanced with increased element spacing to aid users who may have reduced motor control or precision. Furthermore, a clear visual hierarchy was established with minimalistic iconography to reduce cognitive load and enhance more direct navigation. In addition, a comfortable blue palette was utilized for both conveying a sense of trust and ensuring physiological accessibility.

Interaction Convenience: The application made use of a dual-modality interaction by integrating visual and auditory elements to accommodate age-related sensory changes. Visual components contained clear textual instructions, intuitive graphical cues, and progress indicators, while the auditory modality provided detailed vocal instructions and repeatable audio prompts. To address the unique requirements of different tasks while considering the cognitive and physical needs of elderly users, task-specific interface adaptations were implemented. These adaptations included automatic landscape orientation for a wider view and dynamic countdown timers for clear time management cues in specific tasks. In addition, prior to the actual assessment, a Recording Test was carried out to serve as a preparatory measure for users to optimize microphone placement, adjust volume, and verify audio input quality. The application further incorporated sequential task presentation and consistent navigation patterns to effectively break down complex tasks into manageable steps, guide user attention, and reduce the learning curve. These features collectively reinforce the overall interaction convenience for elderly users, harnessing both visual and auditory modalities to support their engagement with the application.

User-Centric Output: The assessment output framework balanced clinical utility with user comprehension. Based on user performance data, the system produced standardized reports, created personalized cognitive profiles, and offered rehabilitation recommendations. For patients, the results were presented in clear, non-technical language, offering insights into their cognitive health and potential improvement strategies. For clinicians or healthcare workers, the reports provided detailed data to guide clinical decision-making. These features enabled long-term cognitive monitoring by tracking changes over time to support ongoing cognitive health maintenance.

#### 2.1.4. Speech Tasks, Feature Extraction, and Classification

The present MCI detection application incorporated three speech–language tasks from our previous work [40]: Picture Description (PD) (includes three figures [44,45,46], details on Supplementary Material SA), Semantic Fluency (SF), and Sentence Repetition (SR). These tasks were designed to be completed in approximately 15 min, with speech transcription and timestamps acquired through ASR. Feature extraction involves two approaches: (1) custom-designed features based on previous work and (2) BERT-based features [47]. A 768-dimensional feature set was extracted from PD task transcriptions using a pre-trained Chinese BERT model. For classification, the application implemented Support Vector Machine (SVM) classifiers for each task, including four task-wise classifiers (two for PD: one using BERT features, another using custom features). Principal Component Analysis (PCA) was used for feature selection in custom feature-based classifiers. Final MCI likelihood is determined by averaging probabilities across all tasks, with equal weighting. A probability threshold of 0.5 is used for MCI classification. This automated approach ensures consistent, objective assessment without manual intervention and improves reliability by analyzing additional lexical and linguistic features from advanced NLP models.

### 2.2. User Engagement Study Procedure

The user engagement study involved 22 participants recruited based on inclusion and exclusion criteria consistent with our previous work [40]. Written informed consent was obtained from all participants, and the study received ethical approval from the Human Research Ethics Committee of Shenzhen Institutes of Advanced Technology, Chinese Academy of Science. Participants completed the MCI screening using our mobile application in a controlled laboratory setting. The block diagram of data collection and workflow is provided in Supplementary Material SB (see Figure S1). An experimenter covertly observed participants’ engagement in two key aspects, using a structured form to record the frequency of these behaviors for each participant during the screening. The two aspects of engagement observed were the following:

(1) Cognitive processing (thinking/analyzing), defined as observable focused or engaged behavior, such as short pauses during speech without obvious distraction or stops to produce words described in filled pauses;
(2) Distraction levels, defined as observable distracted behaviors, such as looking away from the screen, engaging in task-irrelevant speech, or engaging in body movements unrelated to the task.

Immediately following the screening, participants completed a pre-designed questionnaire assessing their overall perceptions of the app’s usability and task difficulty, using 3-point Likert scales and open-ended questions. To provide a validated measure of cognitive performance, each participant underwent a standard MoCA administered by a trained researcher one week after the app screening to minimize potential practice effects. Finally, the collected data from the app screening, engagement observations, questionnaires, along with MoCA scores were analyzed using quantitative and qualitative methods, as described in the Performance Metrics and Data Analysis section.

### 2.3. Performance Metrics and Data Analysis

Technical performance of the MCI screening was evaluated by comparing the automatic assessment results with manual assessment across three speech tasks, and its robustness on a real-world dataset obtained from self-directed, unstructured settings. Standard metrics, including accuracy, precision, recall, and F1-score, were used to assess the performance of the system. For the user engagement analysis, multiple statistical approaches were adopted. First, the Kruskal–Wallis H-test [48] was used to analyze the relationship between users’ overall perception of app difficulty and their cognitive performance (MoCA scores and probability of MCI). Next, to examine the connection between users’ task-specific evaluations and their engagement, both subjective data (self-evaluations of users and observations of experimenters) and objective measures of speech production (task duration, total characters, keywords, and repetitions) were analyzed using the Mann–Whitney U test [49]. Finally, the influence of users’ daily habits and education on their perceptions of the usefulness of the app and willingness to adopt such technologies, were investigated and results were presented using the Kruskal–Wallis H-statistic.

## 3. Results

### 3.1. Participants

The study included data obtained from two distinct groups of participants: the Real-World set and the Survey set. The Real-World set consisted of 12 mobile phone users who completed tasks without any external assistance (three females, nine males), with a mean age of 45.83 years (SD = 10.00 years), mean education level of 16.9 years (SD = 3.87 years), and a mean MoCA score of 26.12 (SD = 1.91). The Survey set included 22 individuals (8 females, 14 males) who participated in the user engagement survey, with a mean age of 60.10 years (SD = 6.48 years), mean education level of 10 years (SD = 3.11 years), and a mean MoCA score of 26.71 (SD = 3.39) *. MoCA scores were available for 17 participants in the Survey set.

### 3.2. MCI Detection Performance

#### 3.2.1. Comparison with Manual Assessment

The present automatic MCI detection system was evaluated against manual assessment benchmarks based on the same dataset. Results (Table 1) demonstrated that the automatic approach yielded comparable or superior performance to manual assessment across all cognitive tasks (F1 ≥ 0.78, Recall ≥0.82).

The task fusion results confirmed the robustness of the present automatic system (F1 = 0.93), closely aligning with manual assessment performance (F1 = 0.95). Notable improvements were observed in the SF task, where the automatic approach substantially outperformed manual assessment (F1: 0.78 vs. 0.64; recall: 0.82 vs. 0.56). The SR task showed slight enhancement in the automatic condition (F1: 0.90 vs. 0.88) while maintaining high precision and recall rates. For the PD task, the BERT-based features achieved comparable performance (F1 = 0.78) to the custom-designed features used in manual assessment (F1 = 0.80), with improved recall (0.86 vs. 0.84) despite a minor precision trade-off. These findings indicate that the present automatic MCI detection system can effectively replace manual assessment, maintaining high performance standards even with the inherent complexities of automated processing.

#### 3.2.2. Real-World Dataset Evaluation

To validate the practical applicability of our automatic MCI detection system beyond laboratory conditions, its performance was evaluated based on a real-world dataset. The task fusion approach demonstrated a reasonable performance (see Table 2) in the real-world settings (F1 = 0.86), despite the observable decrease in results from the controlled environment (F1 = 0.93). However, individual task performance saw substantial declines: SR (F1 = 0.75 vs. 0.90), SF (F1 = 0.44 vs. 0.78), and PD with BERT features (F1 = 0.57 vs. 0.78) (Table 2).

Among individual tasks, SR maintained a relatively robust performance (F1 = 0.75) with perfect recall but reduced precision (0.60). For PD tasks, BERT-based features demonstrated superior performance compared to custom features (F1 = 0.57 vs. 0.46), confirming the robustness of contextual language models in challenging environments. SF showed the most significant performance drop (F1 = 0.44), primarily due to low recall (0.33). The reduced performance likely is due to the combination of a small sample size (*n* = 12), variable environmental conditions, and differences in participant engagement compared to controlled settings.

### 3.3. User Engagement Findings

#### 3.3.1. Overall App Difficulty Perception and Cognitive Performance

To examine the relationship between perceived overall difficulty of the new app (screening process) and cognitive performance (RQ1), the possible correlation between users’ subjective experience of app difficulty and their cognitive status was examined.

As shown in Table 3, the Kruskal–Wallis test revealed no significant relationship between perceived overall difficulty of the app and MoCA score (H = 4.256, *p* = 0.119), as well as probability of MCI (H = 2.387, *p* = 0.303). Among difficulty perception groups (“difficult” *n* = 7, “just ok” *n* = 7, “easy” *n* = 3), those rating the app as “easy” were in marginally better cognitive conditions (MoCA: Median = 29, $P_{25}$ = 29, $P_{75}$ = 29; Prob.MCI: Median = 0.297) compared to the “difficult” group (MoCA: Median = 28, $P_{25}$ = 26, $P_{75}$ = 29; Prob.MCI: Median = 0.456). However, these differences were also not statistically significant. These results contradicted our initial hypothesis (H1) that higher perceived difficulty would be associated with lower cognitive performance and indicate that users’ subjective perception of the overall difficulty of our app is not related to their current cognitive status.

#### 3.3.2. Task-Specific Engagement Patterns

To investigate how users’ task evaluations impact their engagement and therefore speech production (RQ2), the relationship between user perceptions and speech production across three tasks was analyzed. Participants completed each task and provided an immediate evaluation of the tasks through a questionnaire.

**Figure 3:**
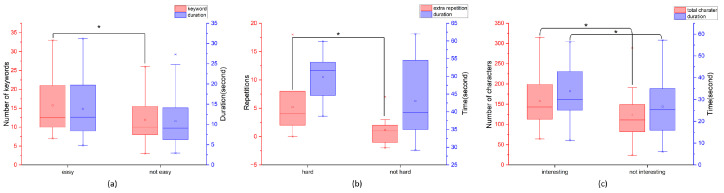
User perception and engagement across different tasks (* *p* < 0.05). Red axes indicate keywords in (**a**), extra repetition in (**b**), and total character in (**c**). Purple axes indicate basically the same thing: the duration or time spent on that specific task.

The primary finding is that positive task engagement corresponded with distinct patterns of speech production, as indicated by users’ self-reported interest and perceived difficulty. In the PD task, which 13 users rated as “most interesting” (comprising 39 sub-tasks), heightened engagement manifested in significantly greater speech output: participants produced more characters and maintained longer speech durations compared to those who found it uninteresting (*p* < 0.05 for both measures; Figure 3c).

Task difficulty also influenced engagement and subsequent speech production patterns. In the SR task, users who reported higher engagement through perceiving it as “hard” produced more speech output, demonstrated by increased repetitions (Median = 4) compared to those rating it “not hard” (Median = 2; *p* < 0.05). These users also spent more time completing the task (Figure 3b). For the SF task, while users who rated sub-tasks as “easiest” (22/66 records) showed higher engagement through producing significantly more keywords, their speech duration did not differ significantly from other users (Figure 3a).

#### 3.3.3. Behavioral Observations and Task Engagement

To complement users’ self-reported engagement, the real-time cognitive behavioral markers through structured experimenter observations during speech production tasks were examined. Observers systematically recorded two key engagement indicators: (1) cognitive processing, manifested through thinking/analyzing behaviors such as focused pauses and word production efforts, and (2) distraction levels, indicated by off-task behaviors such as looking away or irrelevant speech.

**Figure 4:**
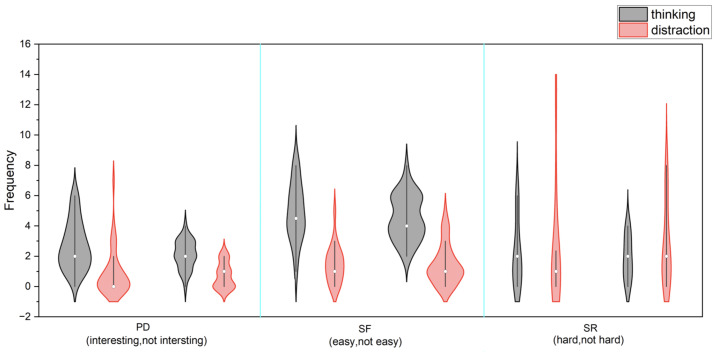
Frequency of observed thinking and distraction behaviors across task perception groups.

Contrary to our expectations that higher self-reported engagement would correspond with more observable cognitive processing and fewer distractions during speech production, the behavioral data showed limited alignment with task perceptions. The primary finding was the consistency of thinking frequency across all perception groups (interesting/not interesting, easy/not easy, hard/not hard) during speech production in all three tasks (Figure 4).

While slight variations in distraction patterns emerged during speech tasks, particularly during the PD task (Median: 0 for “interesting” vs. 1 for “not interesting”) and SR task (Median: 1 for “hard” vs. 2 for “not hard”), the differences did not reach statistical significance. These results suggest that while users’ task perceptions was clearly related to their speech production output, the real-time behavioral engagement during speech production was more uniform than their self-reported engagement would suggest.

#### 3.3.4. Daily Habits, Perceived Benefits, and Technology Adoption

To examine how the daily cognitive exercise habits of the users influenced their perceptions of app usefulness and technology acceptance (RQ3), the questionnaire responses about participants’ daily cognitive activities, perceived benefits of speech–language tasks, and willingness to adopt technology-aided cognitive assessments were analyzed.

The primary finding partially supported our hypothesis that participants’ daily cognitive exercise habits significantly predicted their perception of speech–language tasks’ benefits (H = 9.385, *p* < 0.01; see Table 4). Users who regularly engaged in mental exercises were more likely to recognize the potential benefits of speech tasks for cognitive health monitoring. Interestingly, neither participants’ daily habits nor their education level played a significant role in their openness to technology-assisted cognitive assessment tools (daily habits: H = 0.762, *p* > 0.05; education: H = 7.770, *p* > 0.05). It implies that the willingness to embrace such technology stems from something beyond just cognitive exercise routines or educational background. Despite that, it was found that 86% of the participants expressed their enthusiasm in adopting these systems.

## 4. Discussion

### 4.1. Overview

The automated MCI screening application demonstrated strong technical performance and user engagement, supported by its solid architecture and user-centered design. The client–server architecture, combined with effectively applied user interface principles for the geriatric population, rendered the system able to attain detection performance similar to manual assessment (F1 = 0.93 vs. 0.95) in controlled environments, while ensuring acceptable reliability (F1 = 0.86) in real-world scenarios. The results confirmed the first objective of the present study, to create a dependable and accessible MCI screening tool, although individual task performance showed variability in the practical contexts.

The second objective of exploring user engagement was facilitated by strategic task arrangements and an effective interaction design. The appropriate calibration of task difficulty and the user-friendly interaction design facilitated a high technological acceptance (86%) among users with diverse educational backgrounds. The lack of a link between perceived difficulty and cognitive state, along with the significant beneficial impact of task interest on speech production (*p* < 0.05), suggests that the present speech–language tasks can accommodate users of different cognitive abilities and support the automated approach to creating an engaging and accessible MCI screening tool.

### 4.2. Principal Findings

The proposed automatic MCI detection system demonstrated strong performance when compared with manual assessment across all cognitive tasks. Aligning with the study showing that automated speech analysis can be effective in predicting early cognitive decline [21,22,50], the task fusion approach achieved comparable results (F1 = 0.93) to manual assessment (F1 = 0.95), with remarkable improvements in the SF task (F1: 0.78 vs. 0.64). In addition, the superior performance of BERT-based features over custom features in Picture Description tasks (F1 = 0.57 vs. 0.46) suggests that advanced language models may be particularly valuable for real-world applications.

In real-world situations, a decline in the performance of the system was noted. This could be due to a combination of factors: limited sample size (*n* = 12), fluctuating environmental variables, and variations in participant engagement relative to controlled environments. Despite these challenges, the system exhibited robustness (F1 = 0.86), comparable to existing digital screening tools like Shanghai Cognitive Screening (SCS) with an AUC of 0.838 [18] and another digital tool with an AUC of 0.77 for MCI detection [36].

The strong consistency of the present system with MoCA scores confirms its effectiveness as a digital assessment tool for MCI screening. Integrating the app with traditional methods such as MoCA could provide clinicians with a more comprehensive cognitive profile, potentially enhancing early MCI detection and facilitating timely interventions.

User engagement findings revealed intricate relationships between the perception of overall difficulty and performance outcomes. In contrast to the first hypothesis, perceived app difficulty did not show a significant correlation with cognitive performance, indicating that subjective task difficulty may not serve as a reliable indicator of cognitive status in this instance. Prior studies indicated that performance expectancies influenced actual performance solely in challenging tasks and among individuals with a heightened need for cognition [51]. The absence of a marked correlation between perceived difficulty and cognitive performance in the present study indicates that the difficulty level of the app tasks remained within the cognitive capacities of our general user population.

In relation to the second research question (RQ2) concerning the relationship between users’ evaluations of specific tasks within the app and their engagement levels as well as subsequent speech production, the present findings indicate that subjective task perceptions were positively correlated with both engagement and speech production outcomes, whereas objective engagement markers showed consistency across different perception groups. From a subjective perspective, users’ self-reported task evaluations showed a positive correlation with engagement and speech production outcomes. In the PD task, which many users regarded as “most interesting”, there was an apparent increase in engagement, evidenced by significantly greater speech output. Participants produced more content and sustained longer durations than those who perceived it as uninteresting. In the SR task, users who reported greater difficulty, an indicator of engagement, exhibited increased speech repetitions and allocated more time to the task. In the SF task, despite a less pronounced association between perceptions and engagement, users who rated sub-tasks as “easiest” generated a greater number of keywords, suggesting sustained engagement.

From an objective perspective, the present observational data on real-time engagement markers (cognitive processing and distraction levels) showed that users maintained consistent engagement regardless of their task evaluations. Thinking frequency, defined as observable focused behavior such as short pauses during speech production, remained stable across perception groups during speech production in all tasks. While slight differences emerged in distraction patterns, as indicated by off-task behaviors such as looking away from the screen or task-irrelevant speech, these did not reach statistical significance. This suggests that the app effectively maintained users’ behavioral engagement during the speech production process, independent of their subjective task perceptions.

The third research question (RQ3) examined the impact of users’ daily cognitive exercise practices on the perceived utility of the app and their inclination to adopt similar technology. The research identified a significant correlation between daily cognitive habits and perceived app usefulness (*p* < 0.01), offering important insights for adoption strategies. The 86% technological acceptance rate among participants, irrespective of educational background, indicates extensive use in geriatric health monitoring. Recent research indicated that older individuals are more inclined to utilize mobile health applications when they acknowledge health advantages [52]. This is essential due to the poor diagnostic rates and the necessity for accessible screening instruments in China [6,7]. Highlighting the cognitive health advantages of speech–language exercises and matching the design of the app with user preferences can therefore enhance its perceived usefulness and adoption, irrespective of individual habits or educational background.

### 4.3. Clinical Integration Potential

The app is designed to address early detection of MCI primarily in pre-clinical contexts, especially for large-scale screening initiatives by hospitals, communities, or healthcare facilities. Its standardized, accessible solution and automated nature ensure consistent evaluation metrics, making it particularly valuable for longitudinal cognitive monitoring. Additionally, the app’s self-assessment capability provides individuals with options to manage their cognitive health proactively, potentially leading to earlier clinical consultations when changes are detected. This aligns with our goal on preventive healthcare and early intervention in cognitive decline.

Clinical workflow integration can be considered in the following ways. First, the app can be incorporated into general or annual physical examinations as a routine cognitive screening measure. Second, the objective assessment results can serve as auxiliary measurements to support specialist diagnosis. Finally, long-term cognitive tracking can be applied in clinical settings to capture subtle cognitive changes reflected in language patterns over time, providing specialists with objective data for monitoring disease progression or treatment effectiveness.

### 4.4. Limitations and Future Work

The limited sample size (*n* = 12) for evaluating real-world performance significantly constrains our study and may impact the generalizability of our findings. Due to the limited sample size, the published results—while promising—may not accurately reflect the many characteristics and needs of the broader elderly community. The demographic composition of our sample, especially concerning age range and educational attainment, may not adequately represent the diverse potential consumers who could benefit from our screening tool.

The intrinsic variability of real-world testing situations imposes an additional substantial limitation. Despite achieving reasonable robustness, external factors such as background noise and device discrepancies may introduce variation that affect task performance. In SF, the substantial drop in performance on individual tasks (F1 decreased from 0.78 to 0.44) underscores the difficulties in sustaining assessment performance in uncontrolled settings.

In addition, the examination of user involvement was conducted in laboratory settings. The controlled environment may not accurately reflect the diverse challenges users face in real-life scenarios, including technical difficulties with mobile devices, environmental distractions, or fluctuations in mental or physiological states. The absence of longitudinal data limits our understanding of the evolution of involvement patterns over extended periods of continuous use, particularly among users with diverse cognitive profiles and varying levels of technological familiarity.

Several promising directions for future research can be identified. Emphasis must be placed on executing extensive validation studies across varied populations and contexts, with a particular focus on real-world applications. Long-term follow-up studies may enhance the assessment of the sustainability of user engagement over extended periods. Furthermore, examining the app’s incorporation into current healthcare workflows and assessing its viability as a longitudinal monitoring instrument may improve its clinical applicability. Future research should investigate the influence of caregiver support and social factors on the maintenance of engagement with the application.

## 5. Conclusions

This study reports the development and validation of a speech–language mobile application that addresses two interconnected challenges in MCI screening: assessment performance and user engagement. The automatic screening system exhibited detection performance comparable to manual assessment, while ensuring reliability in practical applications. The application of strategic task organization and user-centered design concepts facilitated the sustained engagement and high technology acceptance among users. Our mobile application provides a viable solution for early detection of MCI on a large scale and therefore drives progress in accessible cognitive healthcare for geriatric populations.

## Figures and Tables

**Table 1:** Comparison of manual and automatic MCI detection performance. BERT refers to features extracted from the BERT NLP model, while Custom refers to custom-designed features from previous work.

	Manual				Automatic			
Tasks	Acc	Pre	Rec	F1	Acc	Pre	Rec	F1
SR	0.90	0.91	0.88	0.88	0.91	0.87	0.94	0.90
SF	0.71	0.74	0.56	0.64	0.79	0.75	0.82	0.78
PD_(Custom)_	0.80	0.80	0.84	0.80	0.80	0.74	0.86	0.80
PD_(BERT)_	-	-	-	-	0.79	0.75	0.82	0.78
Task fusion	0.95	0.97	0.94	0.95	0.94	0.91	0.96	0.93

**Table 2:** MCI detection performance on Real-World dataset.

Task	Accuracy	Precision	Recall	F1
SR	0.67	0.60	1.00	0.75
SF	0.58	0.67	0.33	0.44
PD (Custom features)	0.42	0.43	0.50	0.46
PD (BERT features)	0.50	0.50	0.67	0.57
Task fusion	0.83	0.75	1.00	0.86

**Table 3:** Statistics on influence of task perception on cognitive assessment Outcomes. Overall difficulty perception is categorized as “difficult”, “just ok”, or “easy”. Prob.MCI represents the probability of Mild Cognitive Impairment derived from our task fusion model.

	Overall Perception Median M($P_{25},P_{75}$)			Kruskal–Wallis	
	Difficult (*n* = 7)	Just ok (*n* = 7)	Easy (*n* = 3)	H	*p*
MoCA	28 (26, 29)	25 (22, 28)	29 (29, 29)	4.256	0.119
Prob.MCI	0.456 (0.3, 0.6)	0.470 (0.4, 0.5)	0.297 (0.2, 0.5)	2.387	0.303

**Table 4:** Influence of education and daily habits on benefit valuation and technology acceptance: H-statistic results.

	Education	Daily Habit
Benefit Valuation	H = 7.035	H = 9.385 **
Technology Acceptance	H = 7.770	H = 0.762

** *p* < 0.01.

## Data Availability

The data presented in this study are available on request from the corresponding author (nan.yan@siat.ac.cn). The data are not publicly available due to privacy or ethical restrictions.

## Supplementary Materials

The following supporting information can be downloaded at: https://www.mdpi.com/article/10.3390/bioengineering12020108/s1, SA. Speech Language Tasks; SB. Block Diagram of Data Collection and Workflow; Figure S1: Data Collection and Workflow.
