# Supplementary material for: A Speech-Based Mobile Screening Tool for Mild Cognitive Impairment: Technical Performance and User Engagement Evaluation
Source: Bioengineering (Basel). 2025 Jan 24;12(2):108. doi: 10.3390/bioengineering12020108 (PMC11851810; doi:10.3390/bioengineering12020108)
Supplement: Supplementary file 1 [file bioengineering-12-00108-s001.zip › bioengineering-3416020-supplementary.pdf]

A. Speech Language Tasks

1. Picture Description

In the PD task, participants are asked to describe what they see in the image as thoroughly as possible. Three stimulus images are chosen from studies on impaired speech and diagnosis: 'Cookie theft' from the Boston Diagnostic Aphasia Examination [1], 'Cat rescue' used in conceptualization deficit in aphasia [2], and 'picnic scene' from the Western Aphasia Battery Revised [3]. We add a one-minute restriction to the PD task to examine the temporal aspect of connected speech. This time limit hardly affects speech production results, as the task typically takes less than a minute to complete. Unlike previous studies, we do not intervene during the recording process, even when participants stop speaking before the time limit. This enables fully automated processing, as speaker separation might introduce variations in voiced speech duration without manual verification. To address this, we include a pre-experimental PD task where participants are explicitly informed about the one-minute restriction, asked to speak until the end, and helped to familiarize themselves with the process.

2. Semantic Fluency

In the Semantic Fluency (SF) task, participants are asked to name items from a given category within one minute. Categories include "fruits," "animals," and "Chinese provinces." The SF task limits lexical retrieval to the word level and restricts word selection, requiring additional cognitive functions and memory demands. Participants must mentally rule out non-target words and track previously spoken words while retrieving new ones within the time limit. Additionally, no visual cues are provided, emphasizing information retrieval solely from memory and organizing the task with different cognitive demands from PD.

3. Sentence Repetition

The Sentence Repetition (SR) task consists of 18 sentences arranged in gradually increasing difficulty through lengthened sentences. It relies heavily on working memory and language comprehension. To repeat sentences exactly, participants must memorize them in limited time (one second per character) by words or word blocks, understand their meaning, and reproduce them in the original sequence. Compared to the other two tasks, the SR task directly requires memory recall during speech production, which might capture the memory deficits frequently occurring in patients with MCI.

Reference:

1. Goodglass, H., & Kaplan, E. *Boston diagnostic aphasia examination booklet*. Lea & Febiger, **1983**
2. Hameister, I., & Nickels, L. The cat in the tree–using picture descriptions to inform our understanding of conceptualisation in aphasia. *Language, Cognition and Neuroscience* **2018**, 33(10), 1296-1314.
3. Kertesz, A. Western Aphasia Battery-Revised. *The Psychological Corporation* **2007**.

B. Block Diagram of Data Collection and Workflow

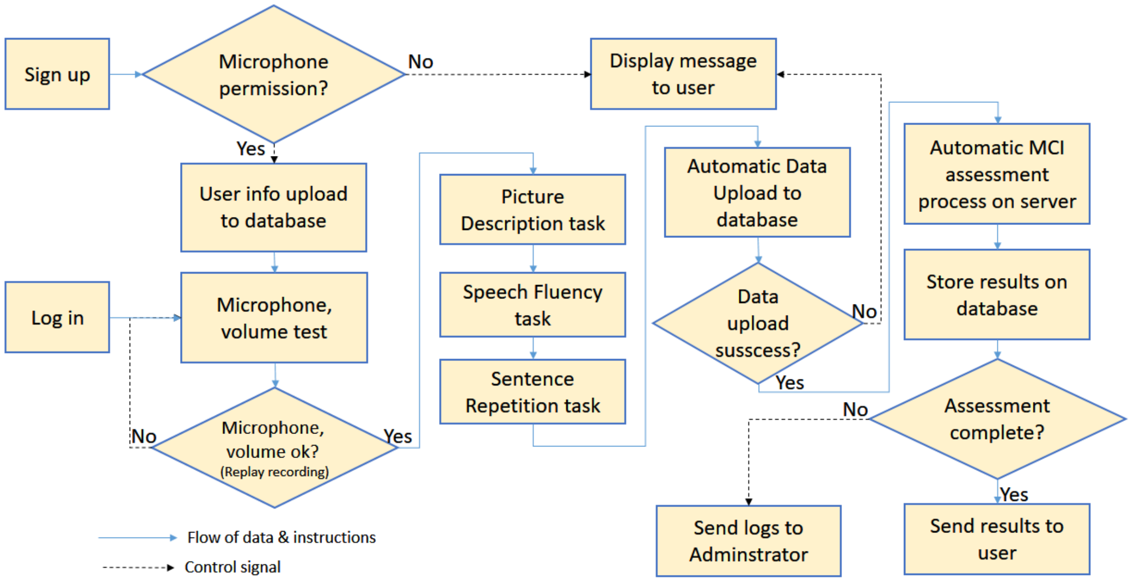

Figure S1. Data Collection and Workflow

Figure B1 presents the comprehensive workflow of the automated speech-based MCI screening system. The system architecture comprises three main functional components:

1. User Authentication and Device Setup

- New users begin with the sign-up process, requiring microphone permission
- Existing users access the system through log-in
- System performs microphone and volume testing to ensure audio quality
- User information is securely stored in the database

2. Speech Task Administration

Three speech tasks are administered sequentially:

- Picture Description task: Users describe a standardized image
- Speech Fluency task: Users generate words within specific categories
- Sentence Repetition task: Users repeat presented sentences

Each task's audio data is automatically uploaded to the database upon completion

3. Data Processing and Results Delivery

- Server conducts automatic MCI assessment using collected speech data
- Results are stored in the secure database
- System generates user reports upon completion
- Administrative logs are maintained for system monitoring

The workflow incorporates error handling mechanisms (dotted lines) for technical issues such as microphone malfunction or data upload failures. Solid lines indicate the primary flow of data and instructions through the system. This architecture ensures systematic data collection, secure processing, and reliable delivery of assessment results.
